# About Methods of Medical Teaching

**Published:** 1881-11

**Authors:** 


### EDITORIAL DEPARTMENT.

About Methods of Medical Teaching.—Much has been said and written about the short-comings of medical education in this country, within the last few years. Most of those who have discussed the subject have ascribed the manifest defects to the short college terms and brief period required for study. Two years' attendance upon lectures is considered by most teachers as utterly insufficient to acquire a sound knowledge of the principles and practice of medicine with its collateral sciences. As a step in advance, the Medical Departments of Harvard, and the University of Pennsylvania, followed by about half a dozen other schools, have lengthened their terms and exacted three years' attendance upon lectures, as prerequisites to obtaining a degree. It is pretty generally admitted, however, that all the other reputable colleges cannot follow this lead without seriously injuring their business prospects. As in this country the State cannot be expected to supply the means of professional education, this must be furnished by private corporations, and hence necessarily becomes largely a matter of business, or perhaps more strictly—money making. An example of the injury which an institution having a large amount invested can sustain, was manifested last year by the diminished classes at Bellevue. As a consequence of the loss incurred by the establishment of the three-years' system, the faculty of this college have returned to the old plan, under which their classes and receipts were so much larger.

But, if we divest ourselves of the notion that it only requires a long time rather than hard study to obtain the needed amount of knowledge to practice medicine successfully, we can at once see that the discussion has been hitherto conducted on a false issue. Harvard and the University of Pennsylvania have distributed the curriculum over three years, but they have done more. They have arranged the subject to be studied in a graded series, placing the elementary branches first in order, and exact an examination on these before the student can proceed with his more advanced studies. He is not allowed to take up a subject like pathology, before he is familiar with physiology, nor will he be expected to understand the course of pregnancy and the mechanism of labor, before he has mastered the anatomy and physiology of reproduction. Hence it appears that the graded system of instruction is the natural one and should be followed in all schools that desire to give their students the advantage of all their opportunities.

Under the system now prevalent, in which the same lectures are repeated to the same classes for two successive years, no regular progression can obtain. A first-course student may, and does, hear the mechanism of labor eloquently expounded by the professor of obstetrics, even before the professor of anatomy has lectured on the topographical anatomy of the pelvis; or he is confused with an account of the complicated process of inflammation, hears about the emigration of leucocytes, and the proliferation of connective tissue cells, before he has learned anything of the anatomy and physiology of the blood, and circulatory system. It may be that a student with "a good head" may gradually evolve some order and connexion out of this chaos, but in the majority of cases, all this mass of knowledge remains only as a sort of hazy and indistinct collection of technical words, of the meaning of which he has no clear or definite knowledge whatever.

Bad as the matter is when the first-course student is assiduous and attentive to his studies, it is infinitely worse when—as is so often the case—he regards his first term as merely a time for frolicking and "having a good time." As a general thing, the first-course student, dreading no responsibility in the shape of an examination at the end of his first term, becomes careless, and if he attends lectures at all, seems to do so in the majority of cases, simply for the purpose of annoying his fellow-students or teachers. At the end of the term he goes back home, perhaps assists his preceptor to the extent of prescribing for a few charity patients during the summer, possibly also makes a spasmodic attack on his Bristowe or Thomas, and returns to college the following winter with the view of obtaining his degree at the end of the second session. His first year in college, instead of having developed habits of industry and order, has rendered him slovenly and careless, wasteful of time and incapable of taking advantage of the opportunities his school may offer. Instead of being able to devote his last few months in school to the orderly acquisition of those practical branches, of which he needs must possess some knowledge in order to practice with success, his mind is worried and confused with the minute details of anatomy and chemistry which he must know to pass the final examination, and which are sure to be forgotten soon after the examination is over That this is only a fair picture of the manner in which the present plan works will be admitted by every candid observer. "Oh, I can read up next summer," or "I'll hear the same thing next winter," is the apology the first-course student makes to himself when it is inconsistent with his personal convenience to attend lectures at certain hours or on certain days.

Considering that so large a proportion of students pass a fairly rigorous examination after such an inconsequent system of pupilage, it must be evident that the details of one-half of the present curriculum of most colleges—and we speak only of reputable institutions—can be mastered by the industrious student who devotes say six months' close attention to them with the aid of a good teacher, and good text-books. Six months' study will not make a man an anatomist, a physiologist, and a chemist ; it takes most men a considerable share of a life-time to become either, but it will enable a student with an average amount of brains well-directed, to acquire sufficient of these branches to enable him to pursue his further studies with profit and even some pleasure. At the end of the six months, his knowledge should be tested by a final examination on these branches, and if he fails to pass in more than one of them, he should not be allowed to proceed to the next grade until he has satisfied the requirements of the first.

The present curriculum could be divided into two courses : the elementary ones—anatomy, physiology, chemistry, materia medica and surgical operations on the cadaver—the first year ; and the higher branches—general and special pathology, and therapeutics, with the various specialties, the second year. A final examination should be given at the end of each term on the subject comprised within the curriculum of that term, to which the student should be urged to pay especial attention. He should also attend all clinics and practical demonstrations, not necessarily for the purpose of learning more about the particular form of diseases illustrated, but to acquire that knowledge of methods of diagnosis and treatment which can only be taught by example and acquired by practice. Most of the colleges in this country have decided that for themselves, at least,the adoption of the three-years'course is impracticable,and that it would destroy their patronage and throw it into the hands of weaker institutions. Bellevue, by reversing her action of the year before, has confessed that she could not afford to take the step. But the plan here suggested is practicable. It would involve no losses, because it would not change the time of the student's attendance upon lectures, except to lengthen the course one month, which is a movement now being slowly made by most schools. On the other hand it would give the student from the day of his matriculation, an ever-present sense of his duties and responsibilities. He would know that at the end of his first term he would have to undergo an examination, failure in which will be as discreditable as if he failed at the end of his second course to get his diploma. It would make students more orderly and attentive at lectures, and more systematic in their studies. It would be more satisfactory to the student himself, while at the same time it would comply to a considerable degree with the just demand for a more thorough education of medical men.

The objection will be urged, that this would be graduating students after attending only one course of lectures,but it is submitted that one course of lectures understood and assimilated by the mind, is worth three or four imperfectly comprehended. Harvard and the schools that followed her lead are doing exactly this, and there is no reason to believe that their graduates stand lower now than when the new system was inaugurated.

An examination at the end of each term would compel the students to carefully review the subjects lectured upon, in their text-book. Under the present system first-course students, notoriously abstain from hard study. They seem to think that their duty is done when they have listened to the lectures, whether they comprehend what was said or not.

The ideas here presented are not put forward as new, but in the recent discussion upon long and multiple terms, the great practical value of examinations—not only as a test of what the student knows but also as a stimulus to exertion—seems to have been quite lost sight of. The man who *knows* his subject, and manifests his knowledge by answering the examiner's questions is the one qualified for a diploma whether he has attended one course of lectures or a dozen. One other reform in medical education seems, however, to be imperative, and it should, perhaps, more properly have been placed first. It is the necessity for a preliminary examination to test the candidate's fitness for entering as a student and profiting by the instruction given. The conditions for admission required by Harvard should be the minimum insisted upon. They are certainly not above the requirements which should be possessed by those who want to understand the lectures upon any scientific subject.

We regard dentistry as a specialty of medicine, and requiring, up to a certain point, instruction in the same branches. When medical and dental training should diverge and become special, we will endeavor to indicate in another article.

